# Basic and clinical immunology – 3012. Per a 10 favors DCs type 2 polarizations by CD40 cleavage and IL-12 suppression in cockroach-sensitive patients

**DOI:** 10.1186/1939-4551-6-S1-P188

**Published:** 2013-04-23

**Authors:** Chhavi Goel, Namita Kalra, BS Dwarakanath, SN Gaur, Naveen Arora

**Affiliations:** 1 Allergy and Immunology Section, Institute of Genomics and Integrative Biology, Delhi, India; 2 Institute of Nuclear Medicine and Allied Sciences, India; 3 Pulmonary Medicine, V.P.Chest Institute, University of Delhi,, Delhi, India

## Background

Cockroach proteases are important risk factors for asthma development in predisposed individuals. The present study investigates the effect of serine protease Per a 10 from *Periplaneta americana* on dendritic cells (DCs) polarization and T-cell responses in cockroach-sensitive patients and healthy controls.

## Methods

Patients of cockroach allergy and healthy controls were selected following American Thoracic Society guidelines. DCs were differentiated from peripheral blood monocytes of patient and control subjects and stimulated with Per a 10. Cells were evaluated for surface markers by flow cytometry and cytokines detected in culture supernatants by ELISA. Autologous DC-T cell co-cultures were done and cytokine levels analyzed in the supernatants.

## Results

DCs from patient and control subjects displayed comparable levels of surface markers CD11c and HLA-DR. CD80 and CD83 were slightly higher on DCs from patient group than controls. CD86 was significantly high on DCs of patient group as compared to the control group. In contrast, CD40 was considerably low on DCs of patients’ group than control group. Within the patients’ group, CD40 was low on DCs pulsed with proteolytically active Per a 10 than inactive Per a 10. This was corroborated by two-fold decrease in IL-12 production in DC cultures pulsed with active Per a 10 as compared to inactive Per a 10 (*P*<0.05). These active Per a 10-pulsed DCs also showed elevated secretions of IL-5 and IL-6 than those by inactive Per a 10-pulsed DCs (*P*<0.05). Active Per a 10-stimulated DCs also induced significantly (*P*<0.05) low IL-12 and increased levels of IL-4, IL-5 and IL-6 by T cells as compared to inactive Per a 10. Between patient and control groups, low IL-12 and high IL-5, IL-6 were observed in DC cultures of patient than control group (*P*<0.05). Further, Per a 10-stimulated DCs from patients’ induced significantly (*P*<0.05) increased secretions of IL-4, IL-5, IL-6 and low IL-12 by T cells in comparison to those from healthy controls.

## Conclusions

Patients’ DCs on stimulation with Per a 10 favors type 2 polarizations by increase in CD86 expression and low IL-12 secretion. This may be mediated by CD40 cleavage due to proteolytic activity of Per a 10.

